# Supplementary material for: A high-risk gut microbiota configuration associates with fatal hyperinflammatory immune and metabolic responses to SARS-CoV-2
Source: Gut Microbes. 2022 May 15;14(1):2073131. doi: 10.1080/19490976.2022.2073131 (PMC9116414; doi:10.1080/19490976.2022.2073131)
Supplement: Supplemental Material [file KGMI_A_2073131_SM9585.zip › Supplementary Figures.pdf]

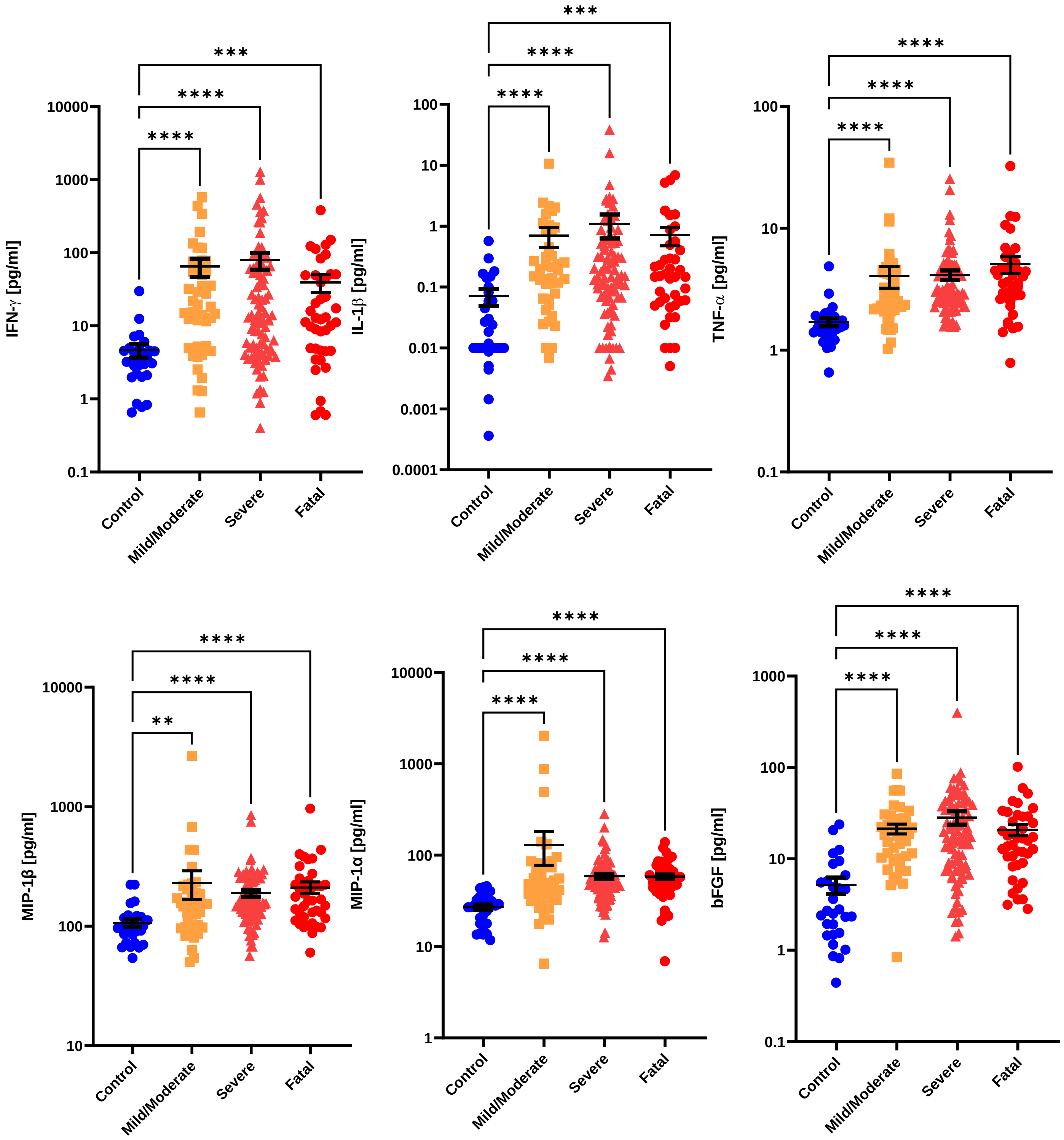

Figure S1. Serum cytokine levels.
Results are expressed as mean and standard deviation. Differences between groups are calculated using the Kruskal-Wallis test and Dunn's multiple comparison test (*p<0.05, **p<0.01, ***p<0.001, ****p<0.0001).

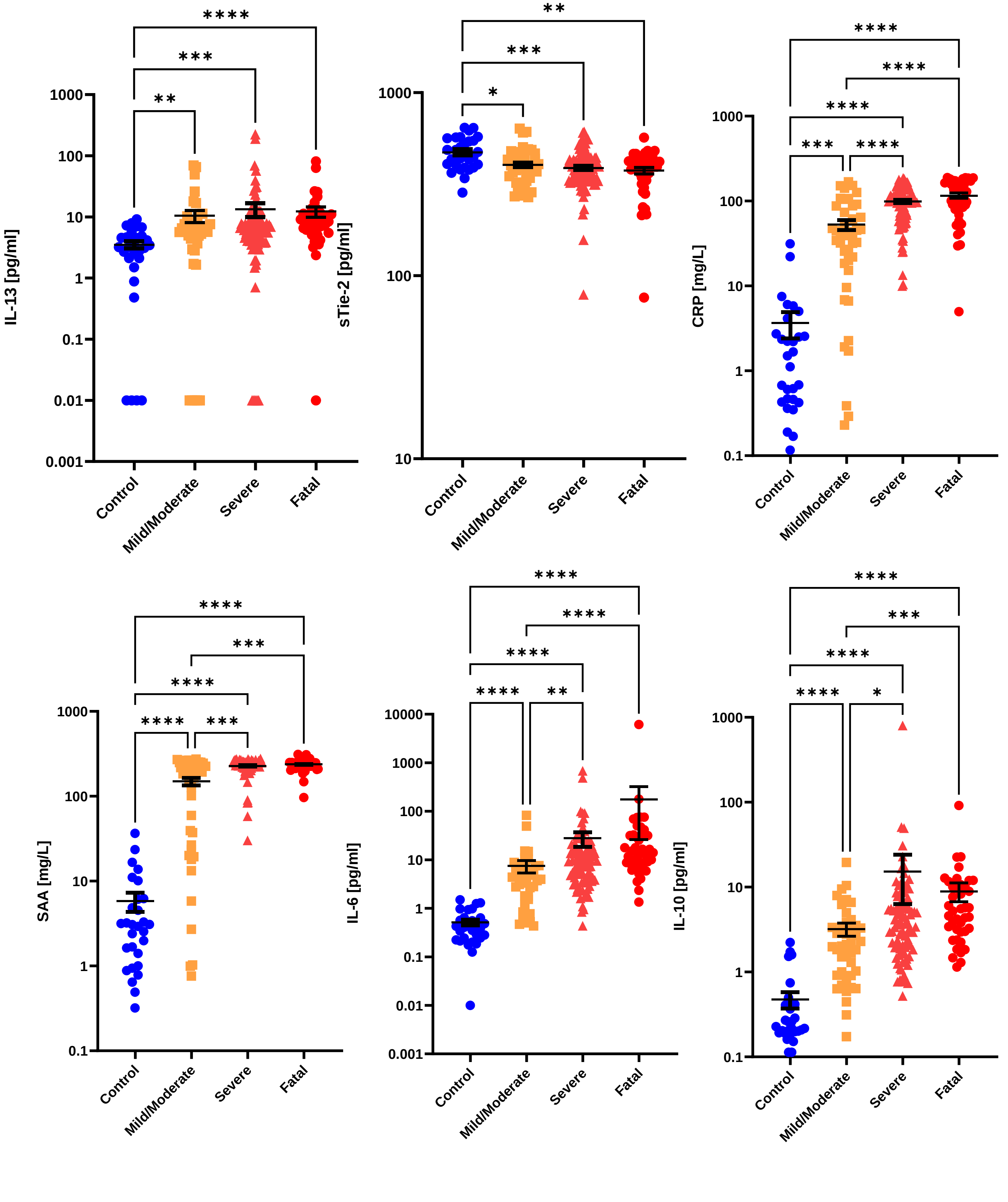

Figure S1. Serum cytokine levels (Continued)

Results are expressed as mean and standard deviation. Differences between groups are calculated using the Kruskal-Wallis test and Dunn's multiple comparison test (*p<0.05, **p<0.01, ***p<0.001, ****p<0.0001).

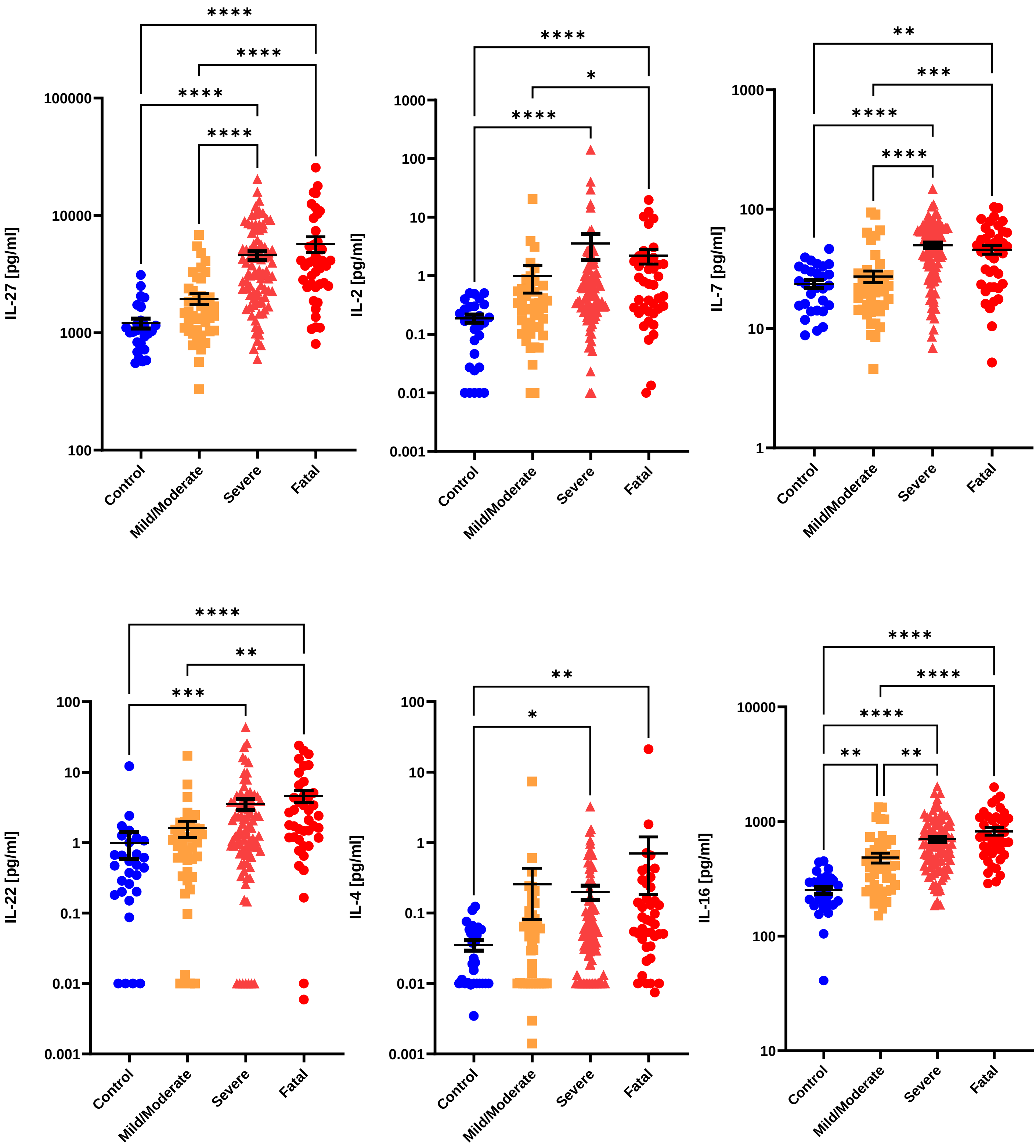

Figure S1. Serum cytokine levels (Continued)

Results are expressed as mean and standard deviation. Differences between groups are calculated using the Kruskal-Wallis test and Dunn's multiple comparison test (*p<0.05, **p<0.01, ***p<0.001, ****p<0.0001).

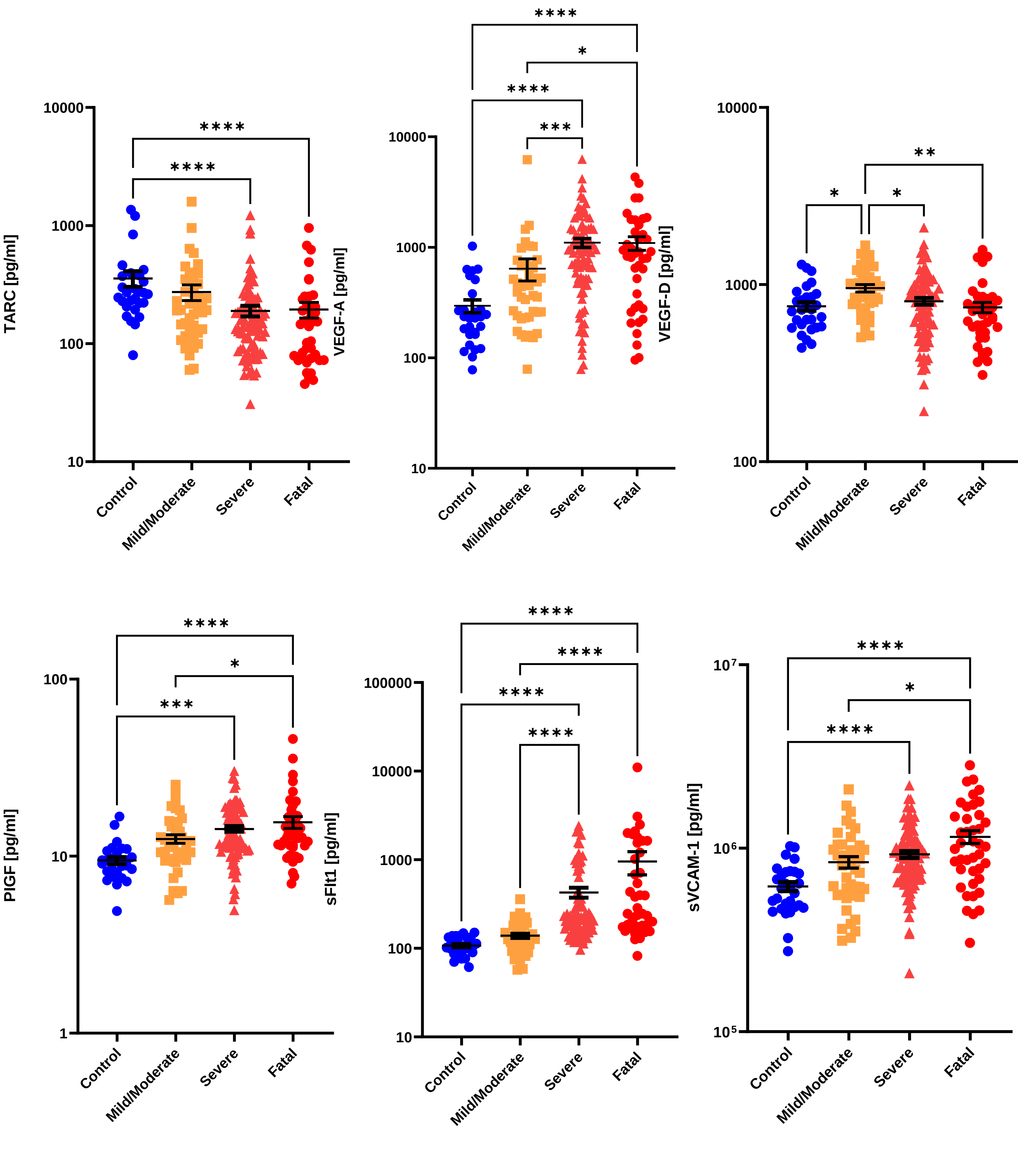

Figure S1. Serum cytokine levels (Continued)

Results are expressed as mean and standard deviation. Differences between groups are calculated using the Kruskal-Wallis test and Dunn's multiple comparison test (*p<0.05, **p<0.01, ***p<0.001, ****p<0.0001).

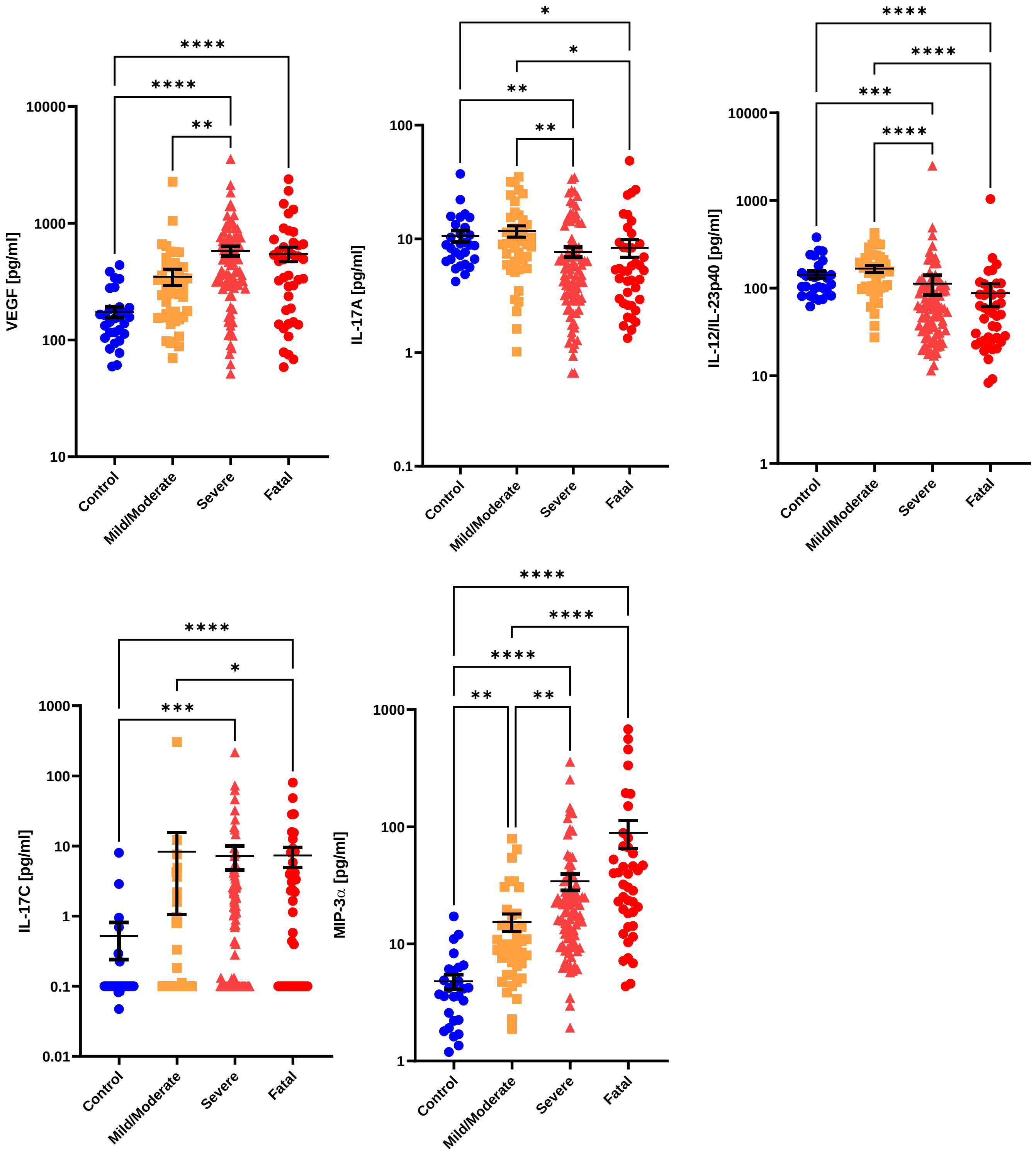

Figure S1. Serum cytokine levels (Continued)

Results are expressed as mean and standard deviation. Differences between groups are calculated using the Kruskal-Wallis test and Dunn's multiple comparison test (*p<0.05, **p<0.01, ***p<0.001, ****p<0.0001).

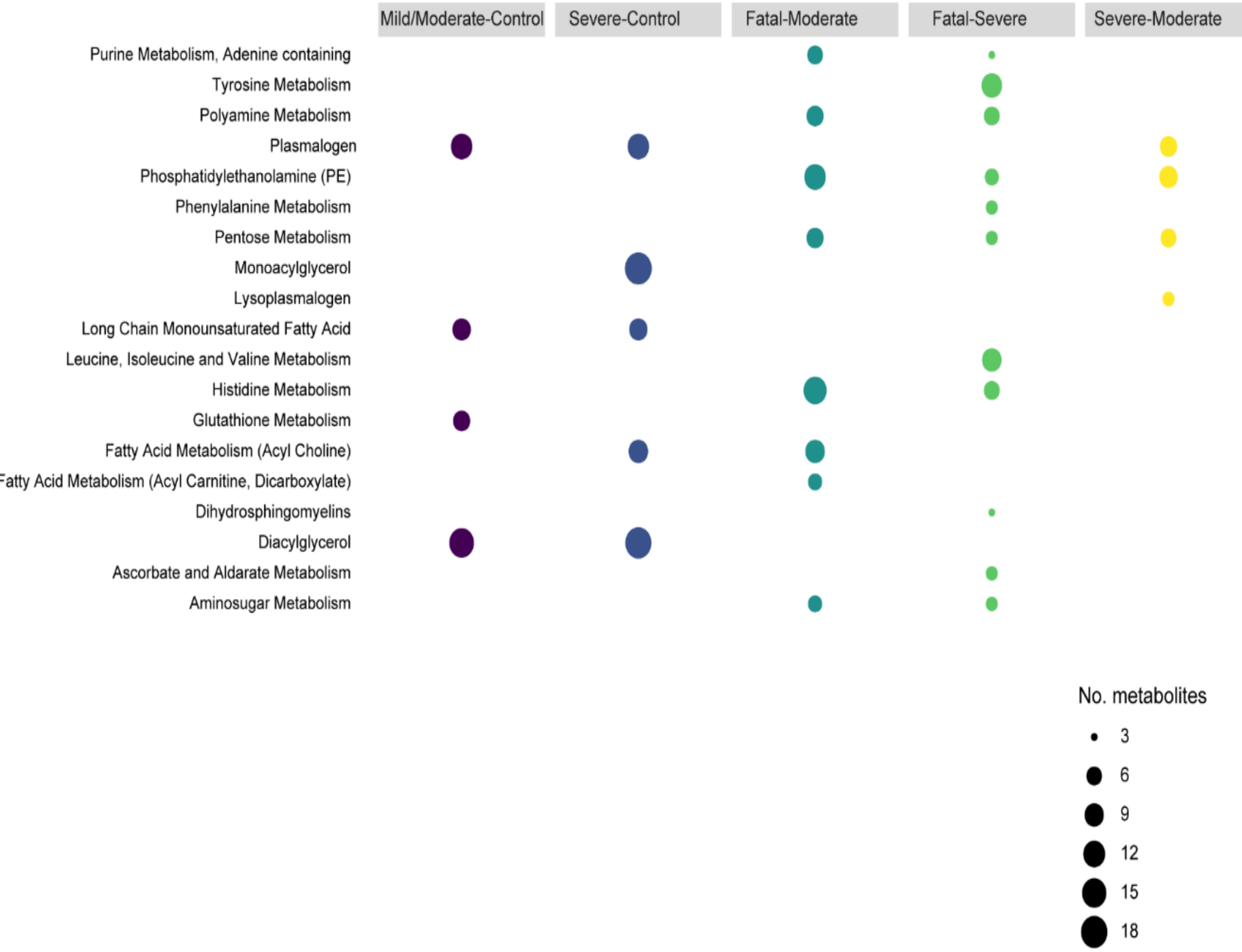

Figure S2. Metabolite set enrichment analysis.
Using Metabolon terms (gseapy, FDR < 0.2), bubble size represents the number of metabolites found in each pathway. Color is specific to each comparison.

a

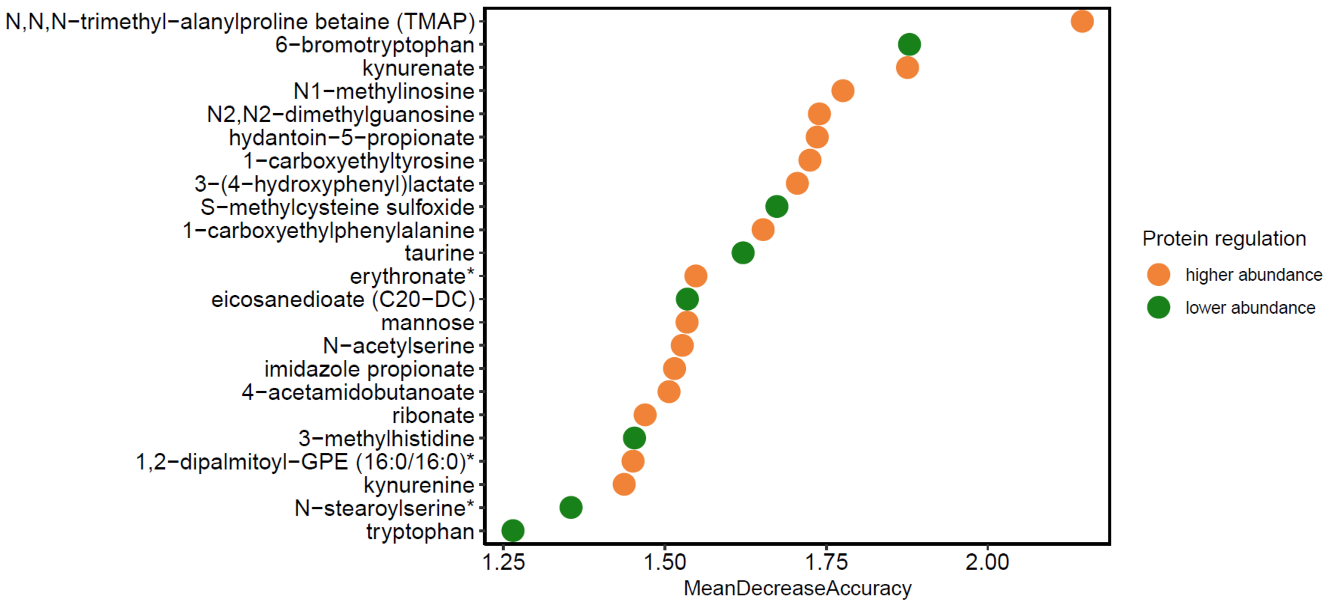

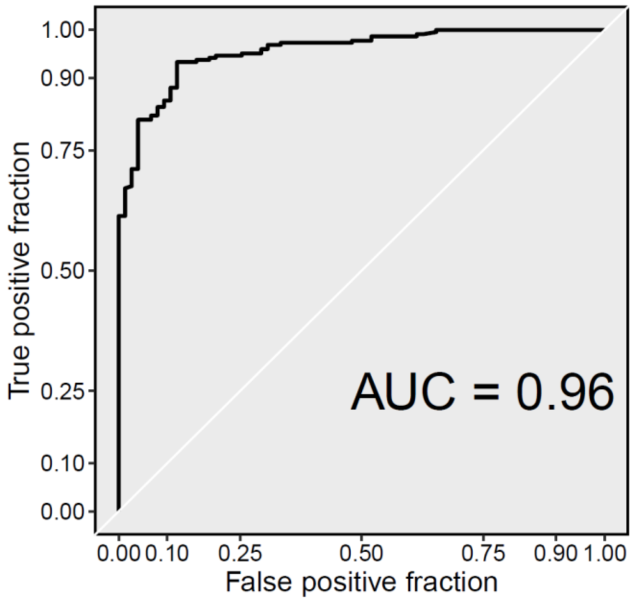

Random forest analysis of Mild/Moderate compared to Severe

Figure S3. Random forest analysis of serum metabolites.
The metabolite features and AUC curves for random forest analysis of COVID-19 patients with mild/moderate disease compared to those with severe disease (a); COVID-19 patients with a fatal outcome compared to those with mild/moderate disease (b); COVID-19 patients that survive following severe disease compared to those that don't survive following severe disease (c).

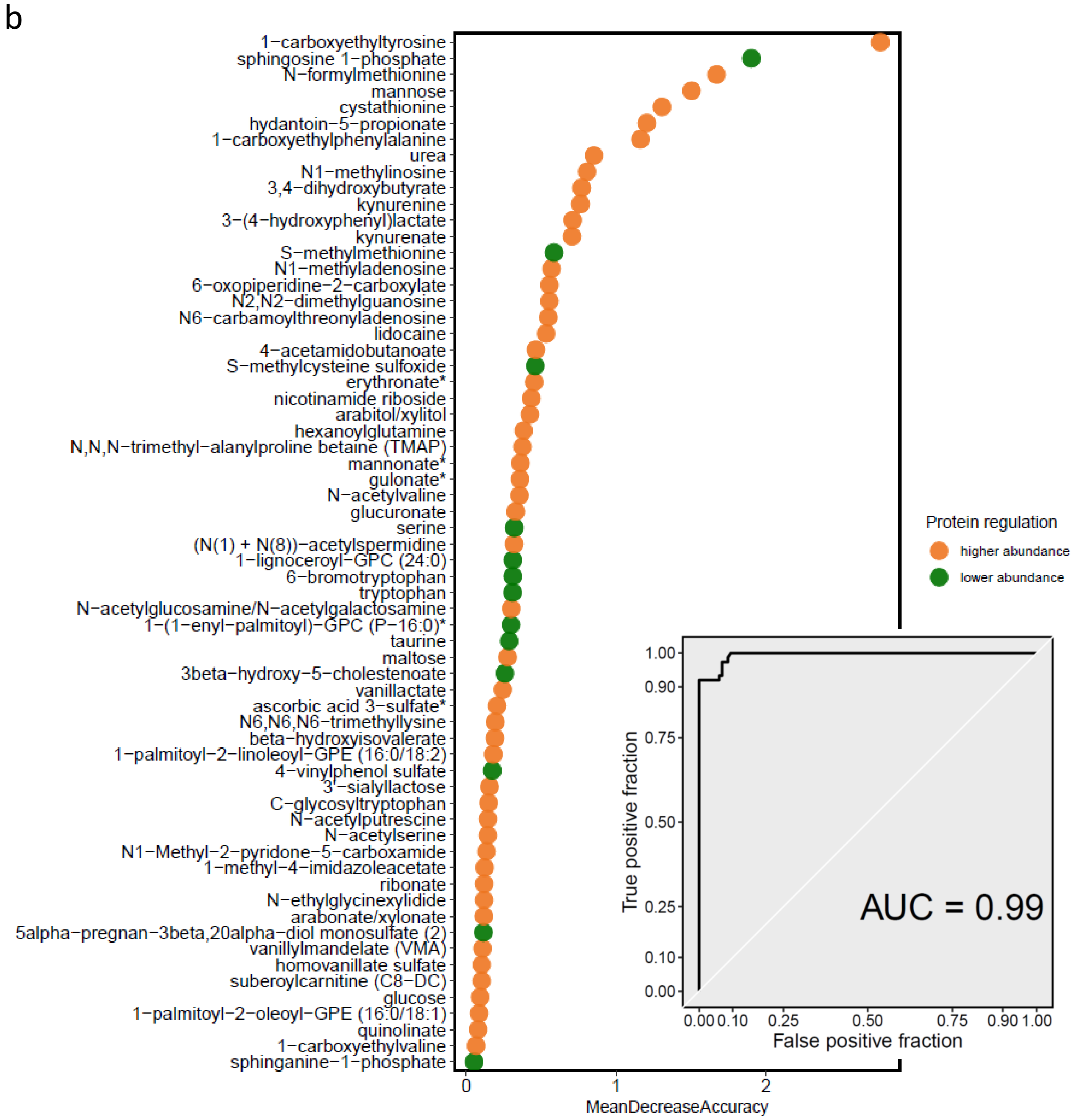

Random forest analysis of fatal compared to Mild/Moderate

Figure S3. Random forest analysis of serum metabolites (continued)
The metabolite features and AUC curves for random forest analysis of COVID-19 patients with mild/moderate disease compared to those with severe disease (a); COVID-19 patients with a fatal outcome compared to those with mild/moderate disease (b); COVID-19 patients that survive following severe disease compared to those that don’t survive following severe disease (c).

c

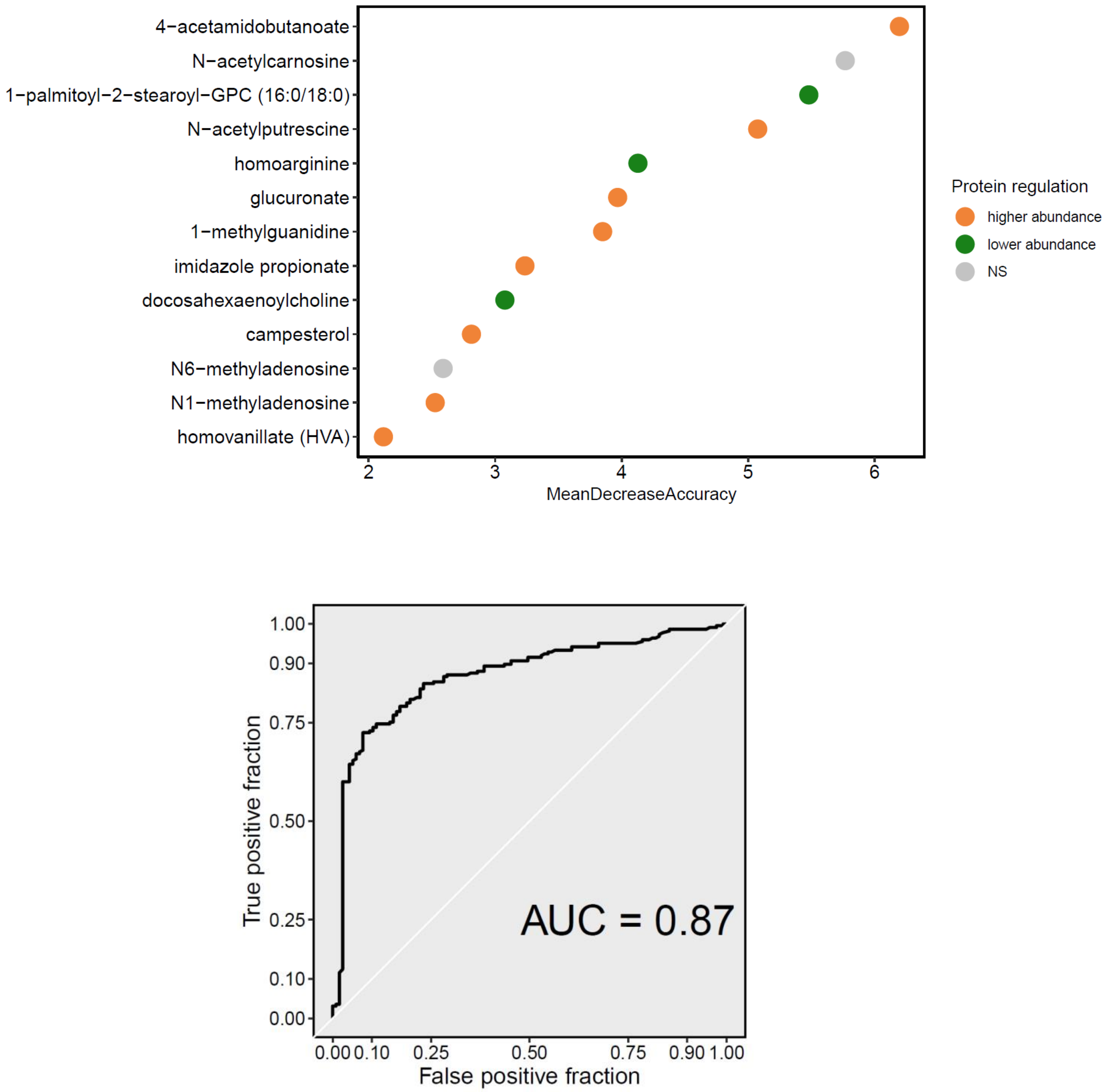

Random forest analysis of fatal compared to severe

Figure S3. Random forest analysis of serum metabolites (continued)
The metabolite features and AUC curves for random forest analysis of COVID-19 patients with mild/moderate disease compared to those with severe disease (a); COVID-19 patients with a fatal outcome compared to those with mild/moderate disease (b); COVID-19 patients that survive following severe disease compared to those that don’t survive following severe disease (c).

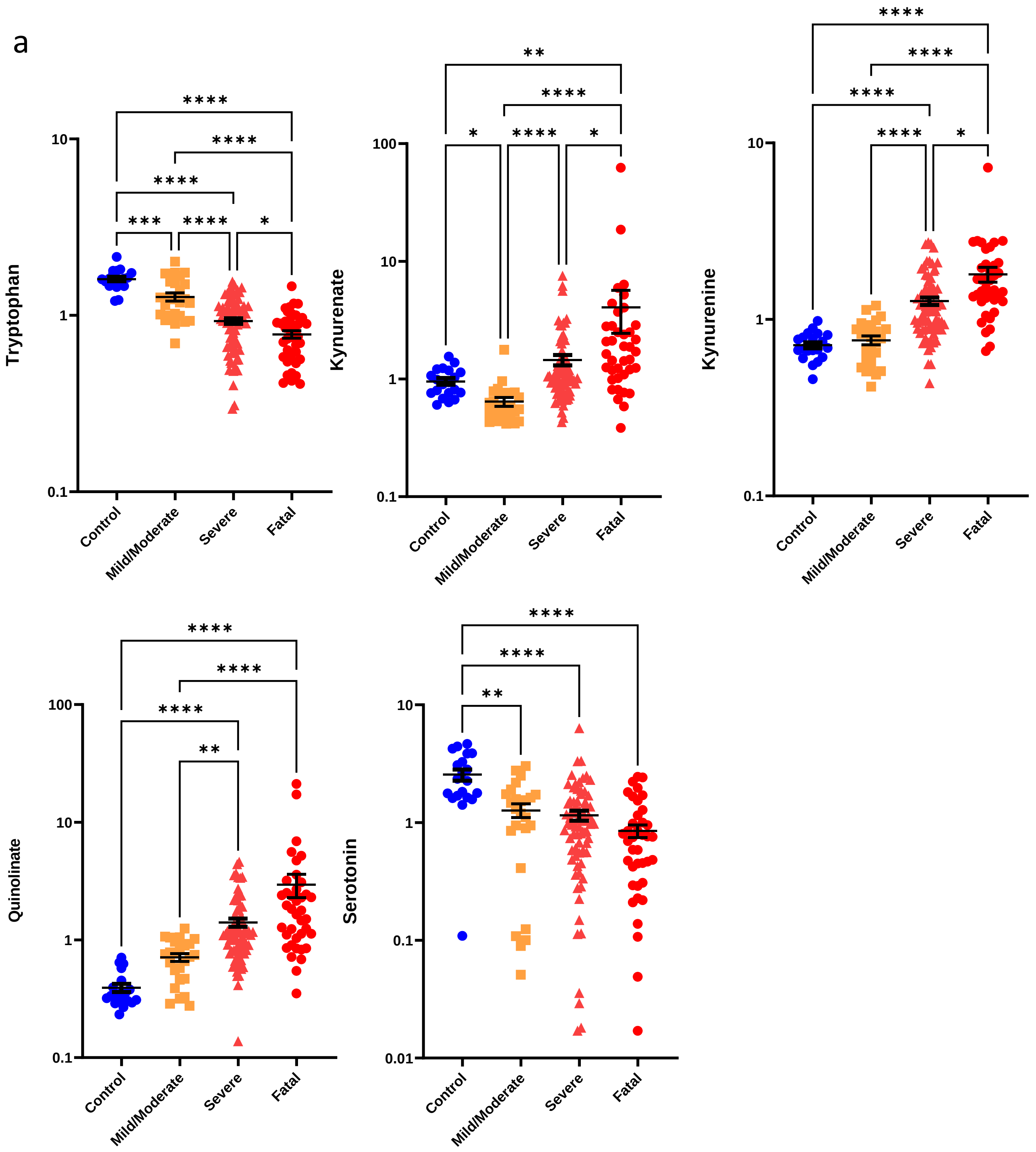

Figure S4. Serum microbial metabolites.
Representative examples of metabolites generated by host metabolism of tryptophan (a). Selected examples of serum levels of microbial metabolites due to tryptophan metabolism (b) or SCFAs (c). Serum citrulline levels (d). Results are expressed as mean and standard deviation. PCA plot illustrates the differences in serum metabolites associated with microbial metabolism (e). Differences between groups are calculated using the Kruskal-Wallis test and Dunn's multiple comparison test (*p<0.05, **p<0.01, ****p<0.0001).

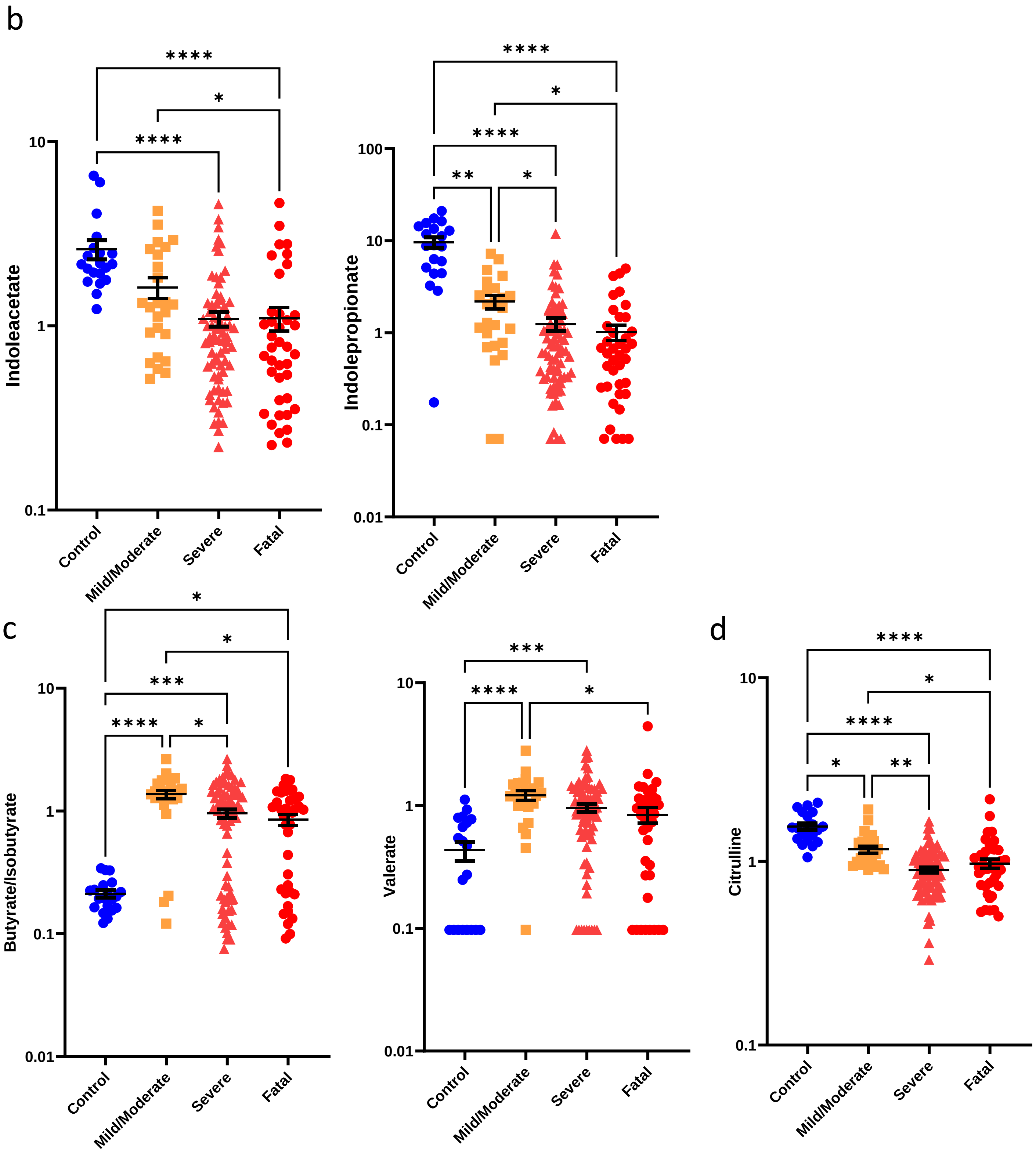

Figure S4. Serum microbial metabolites (continued)
Representative examples of metabolites generated by host metabolism of tryptophan (a). Selected examples of serum levels of microbial metabolites due to tryptophan metabolism (b) or SCFAs (c). Serum citrulline levels (d). Results are expressed as mean and standard deviation. PCA plot illustrates the differences in serum metabolites associated with microbial metabolism (e). Differences between groups are calculated using the Kruskal-Wallis test and Dunn's multiple comparison test (*p<0.05, **p<0.01, ****p<0.0001).

e

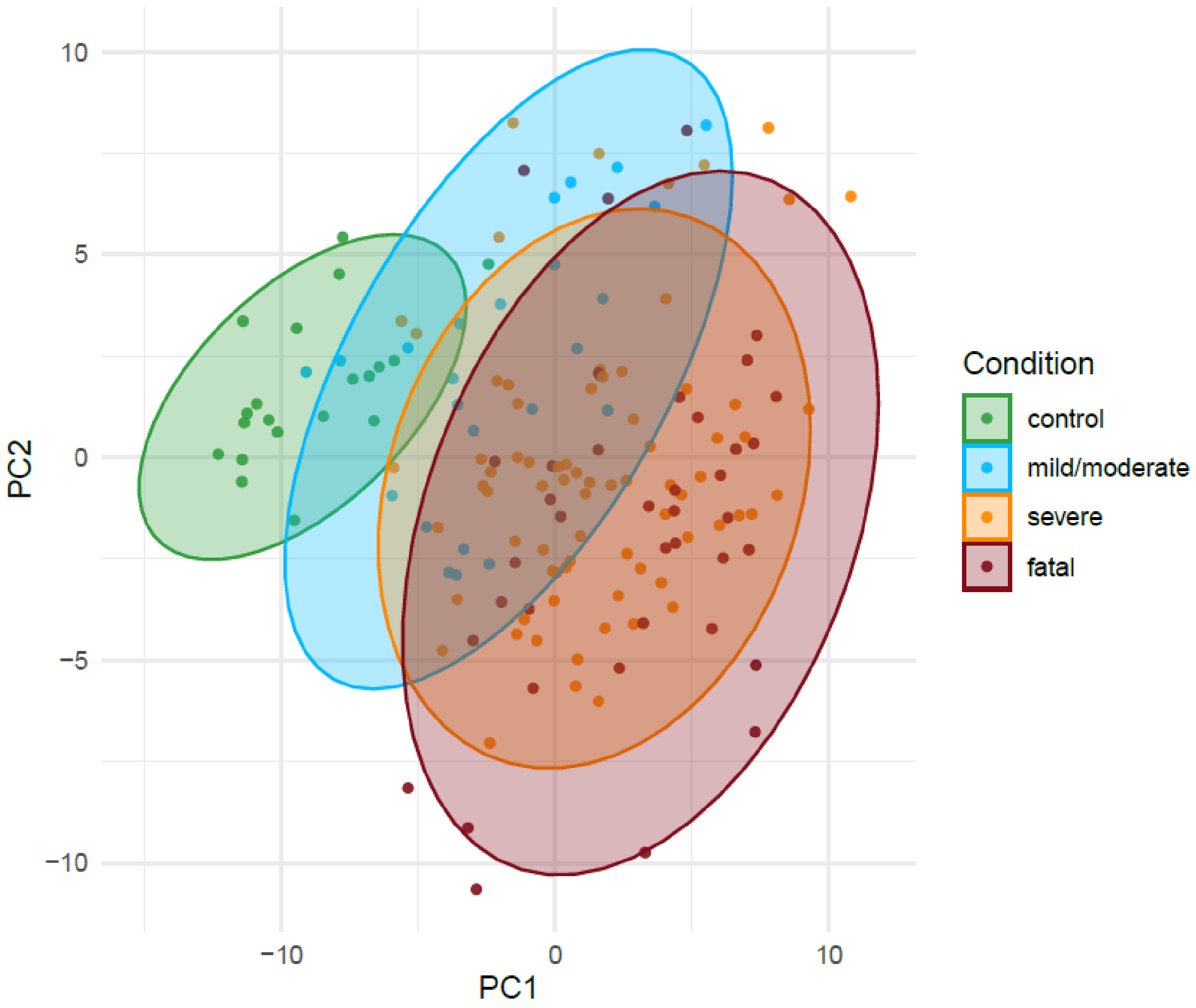

Figure S4. Serum microbial metabolites (continued)
Representative examples of metabolites generated by host metabolism of tryptophan (a). Selected examples of serum levels of microbial metabolites due to tryptophan metabolism (b) or SCFAs (c). Serum citrulline levels (d). Results are expressed as mean and standard deviation. PCA plot illustrates the differences in serum metabolites associated with microbial metabolism (e). Differences between groups are calculated using the Kruskal-Wallis test and Dunn's multiple comparison test (*p<0.05, **p<0.01, ****p<0.0001).

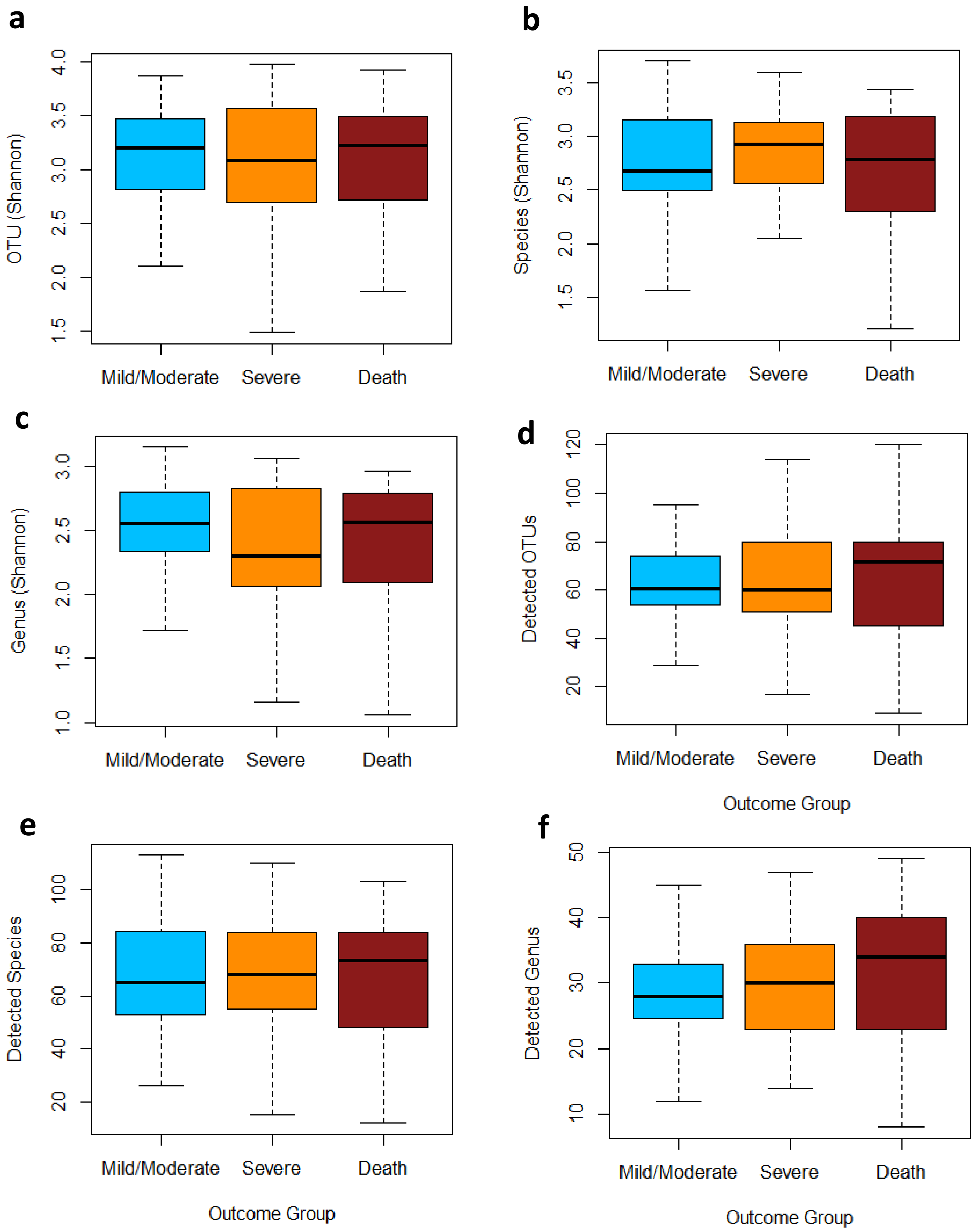

Figure S5. Gut microbiome alpha diversity.

Boxplots showing the variation of the Shannon Diversity and Detected taxa for the gut microbiome profiles for the three outcome groups at OTU (a and d), Species (b and e) and Genus (c and f) levels.

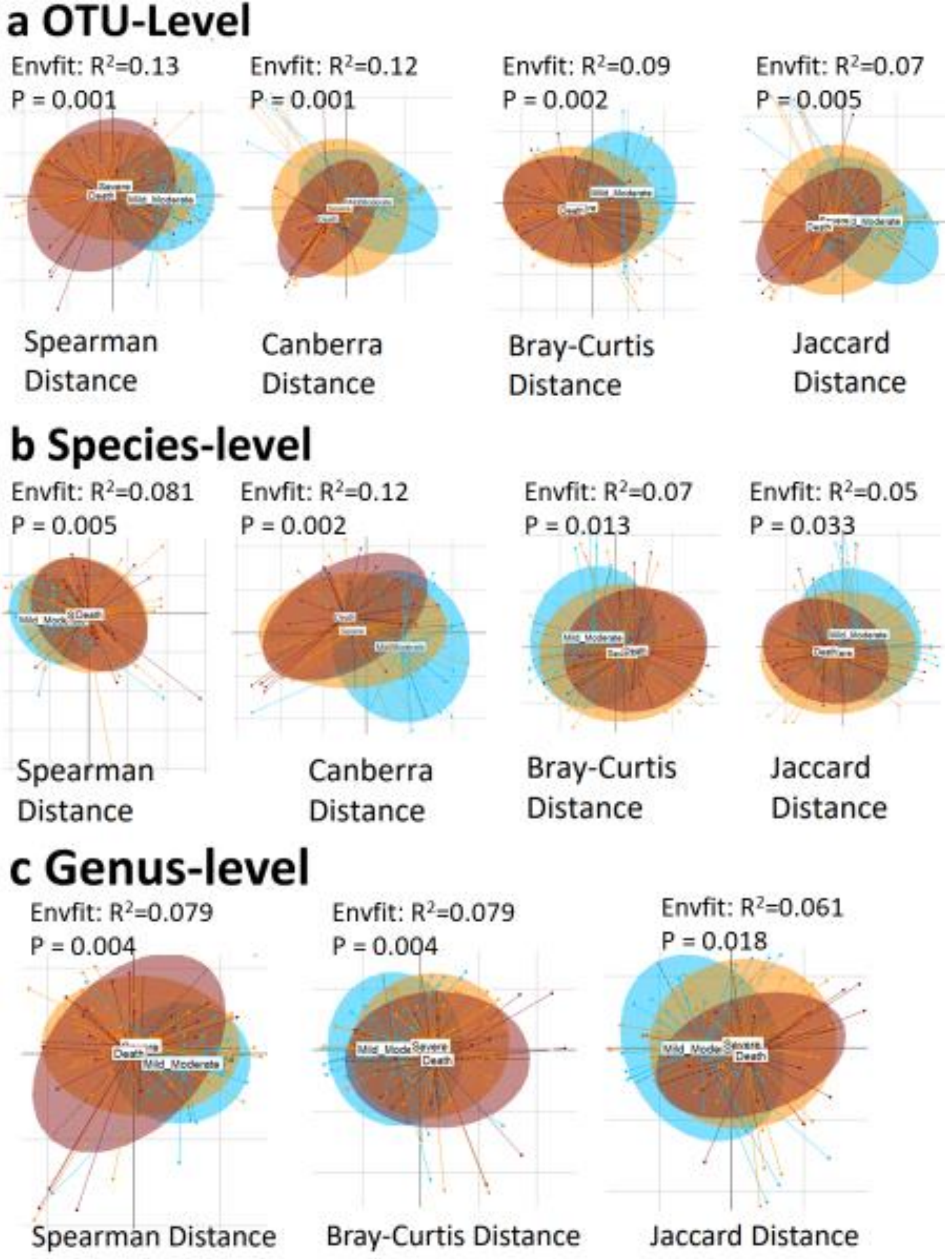

Figure S6. Gut microbiome beta diversity.
Principal coordinate analysis showing the resolution of the gut microbiome profiles from the 99 patients belonging to the three outcome groups at (a) OTU and (b) Species level, obtained using four different distance measures. (c) Principal coordinate analysis showing the resolution of the gut microbiome profiles from the 99 patients belonging to the three outcome groups at the genus level obtained using the Spearman, Bray-Curtis and Jaccard distance measures.

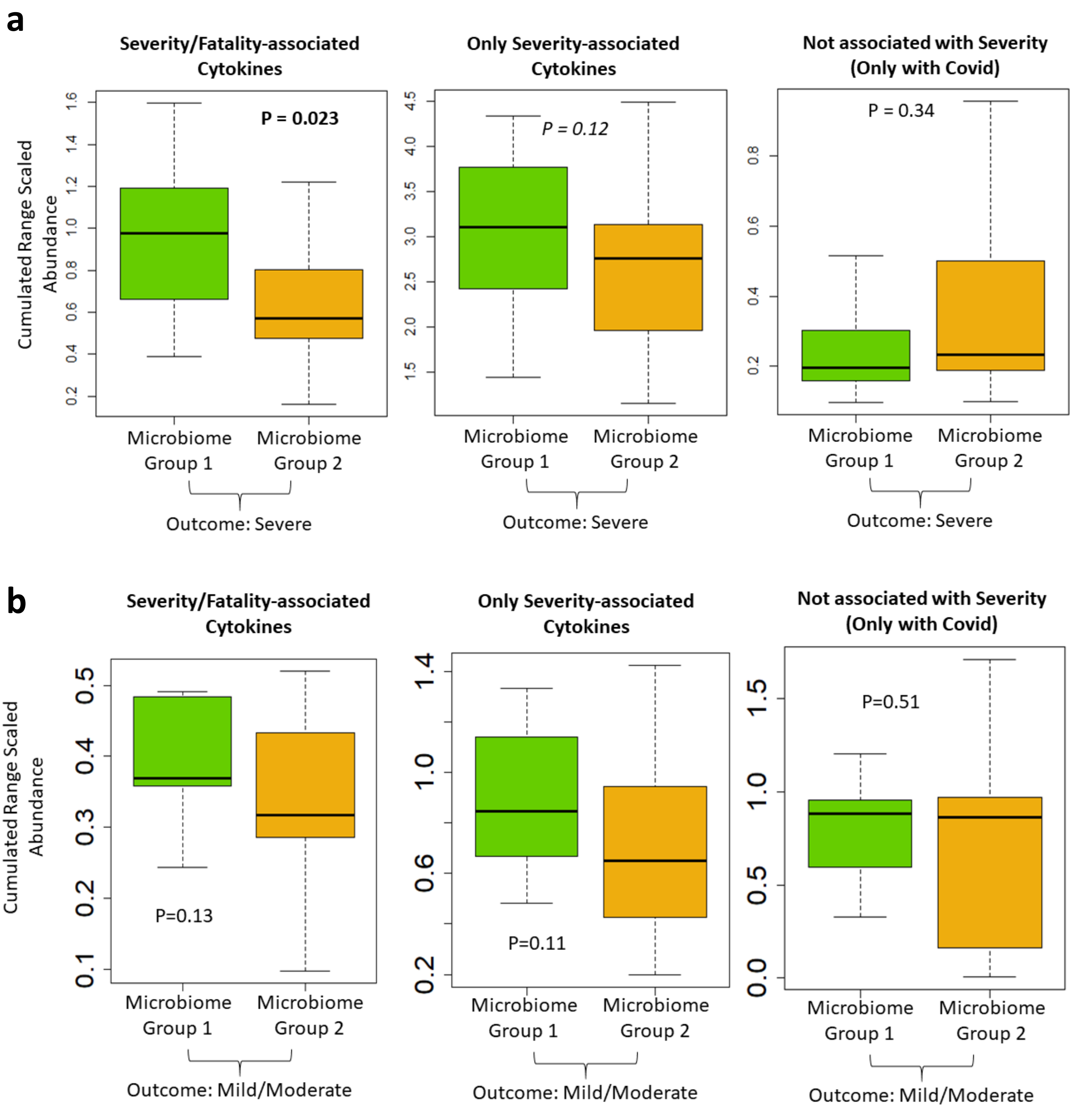

Figure S7. Cytokine levels associated with Microbiome Groups.

(a) Boxplot showing the differences in the cumulated range-scaled levels of the three groups of elevated cytokines between surviving patients with severe symptoms who had a high-risk MicrobiomeGroup1 and those patients with severe symptoms who were classified to the low-risk MicrobiomeGroup2. The p-values of the Mann-Whitney tests obtained for the comparisons within the three groups of cytokines are indicated. Each cytokine level was range-scaled across patients to a value between 0 and 1. For each patient, the range-scaled values of all cytokines within the same group were then cumulated by adding the corresponding range-scaled values obtained for the given patient. (b) Boxplot showing the differences in the cumulated range-scaled levels of the elevated cytokine levels between patients with mild/moderate disease who had a high-risk MicrobiomeGroup1 and those patients with mild/moderate disease who were classified to the low-risk MicrobiomeGroup2.

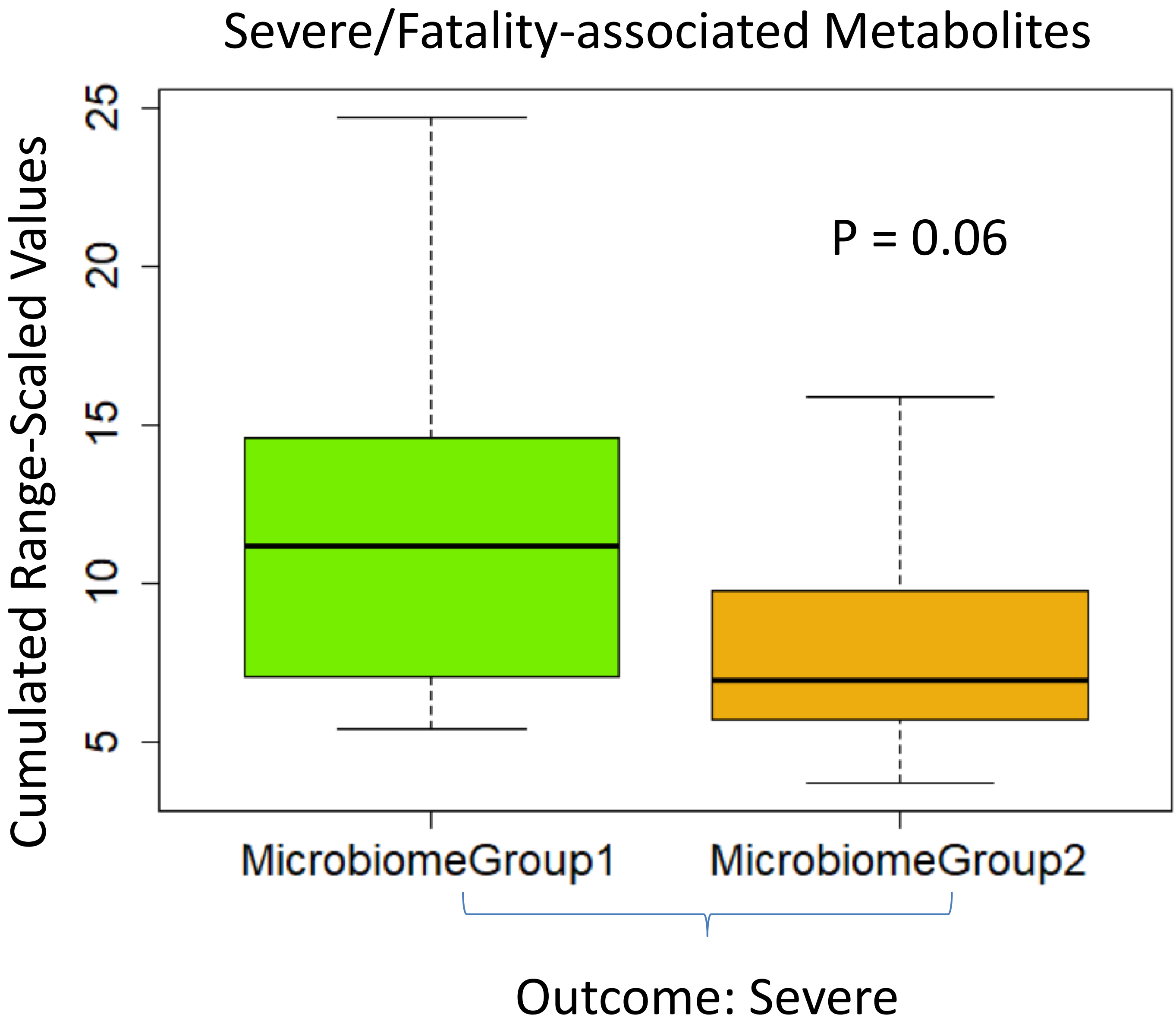

Figure S8. Metabolite levels associated with Microbiome Groups.
Boxplot showing the differences in the cumulated range-scaled levels of the elevated metabolites between surviving patients with severe symptoms who had a high-risk MicrobiomeGroup1 and those patients with severe symptoms who were classified to the low-risk MicrobiomeGroup2. The p-values of the Mann-Whitney tests obtained for the comparisons are indicated. Each metabolite level was range-scaled across patients to a value between 0 and 1. For each patient, the range-scaled values of all metabolites within the same group were then cumulated by adding the corresponding range-scaled values obtained for the given patient.

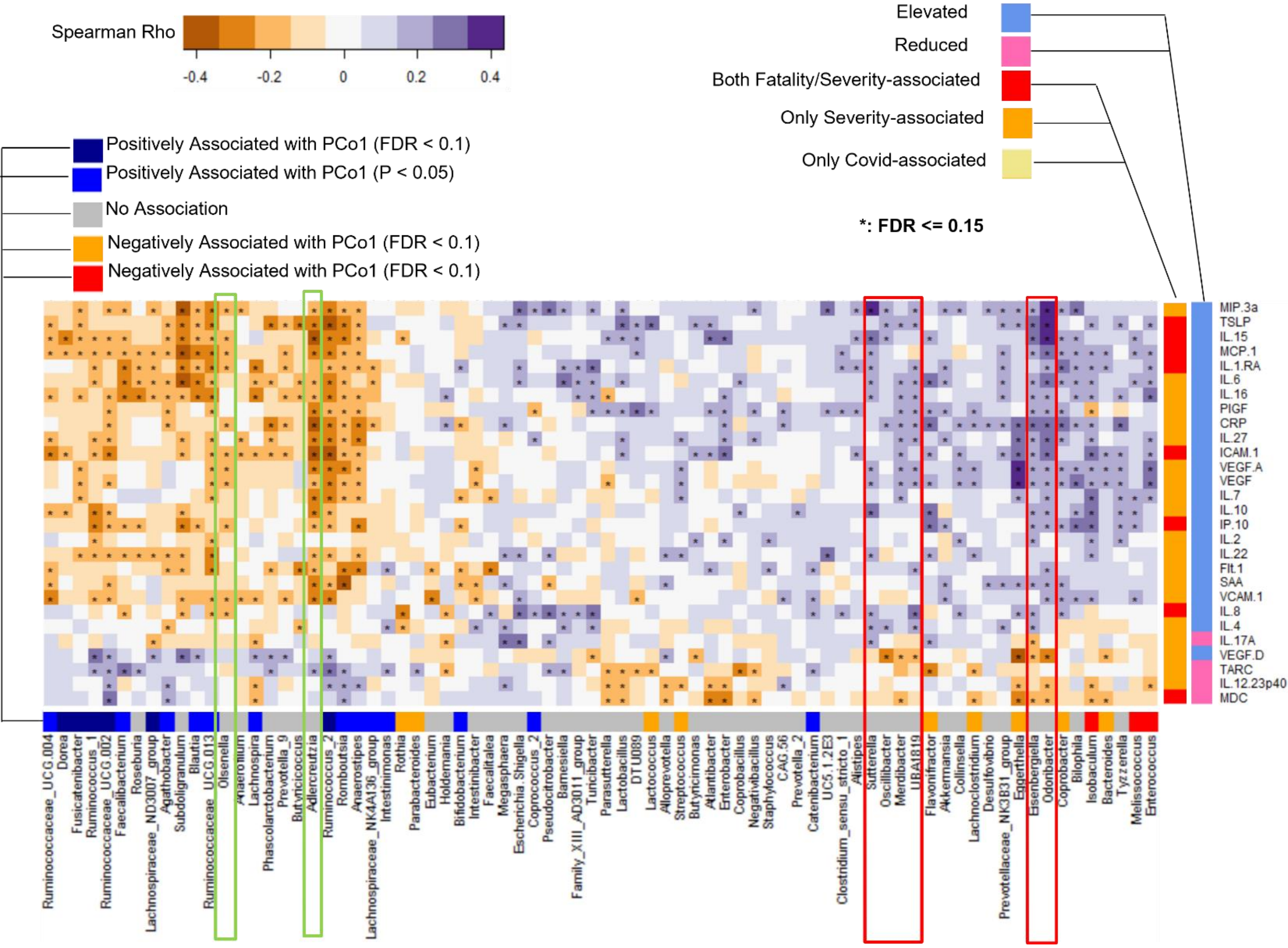

Figure S9. Bacterial genera correlate with circulating inflammatory mediators.

Heatmap showing the Spearman correlations between the 73 genus-level markers (detected in at least 5 of the 99 patients and showing association with FDR≤0.15 with at least one of the severity/fatality-associated cytokines) and 28 severity/fatality-associated cytokines. The groups of the different cytokines and their direction of change (elevated or reduced) with severity/fatality are also indicated in specific colors. Also indicated are the patterns of the various genus-level markers with PCo1 that resolves the two microbiome groups (positive PCo1 with MicrobiomeGroup2). The genera whose associations are indicated in red boxes are those that did not show any associations with either of the two Microbiome configurations (or groups) in terms of their association with PCo1, but independently show association with an inflamed host phenotype. The genera in green boxes show the opposite trends with the inflamed phenotype.

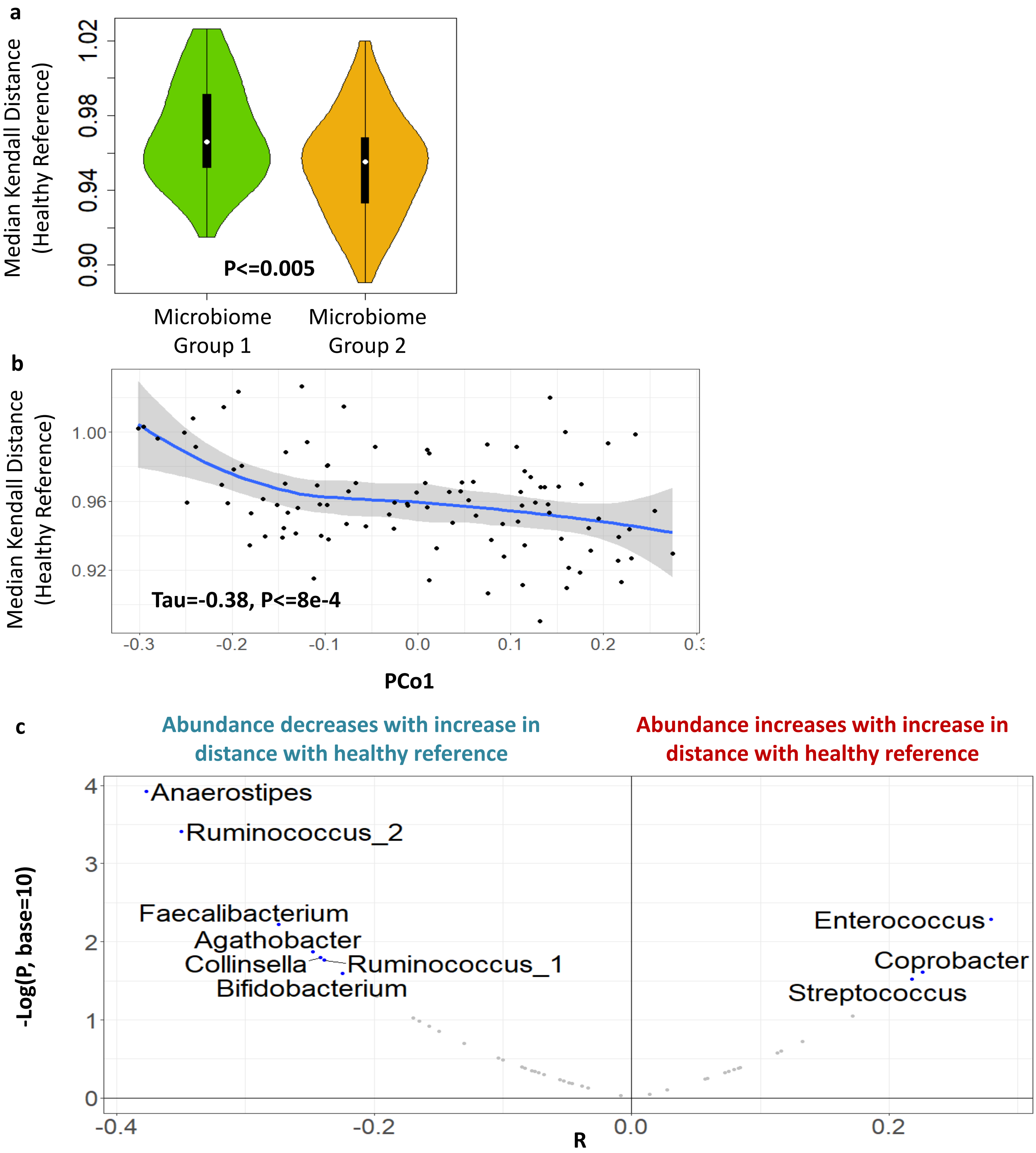

Figure S10. Microbiome comparison to a healthy reference dataset
(a) shows the median Kendall distance to the healthy reference dataset. (b) Shows the PCo1, where the PCo1 decreases (that is the microbiome acquires the high-risk configuration), the distance from the healthy reference control significantly increases. (c) Genera are shown that are associated with decreased or increased distance to the healthy reference control dataset (only those with Q<=0.15 are indicated).

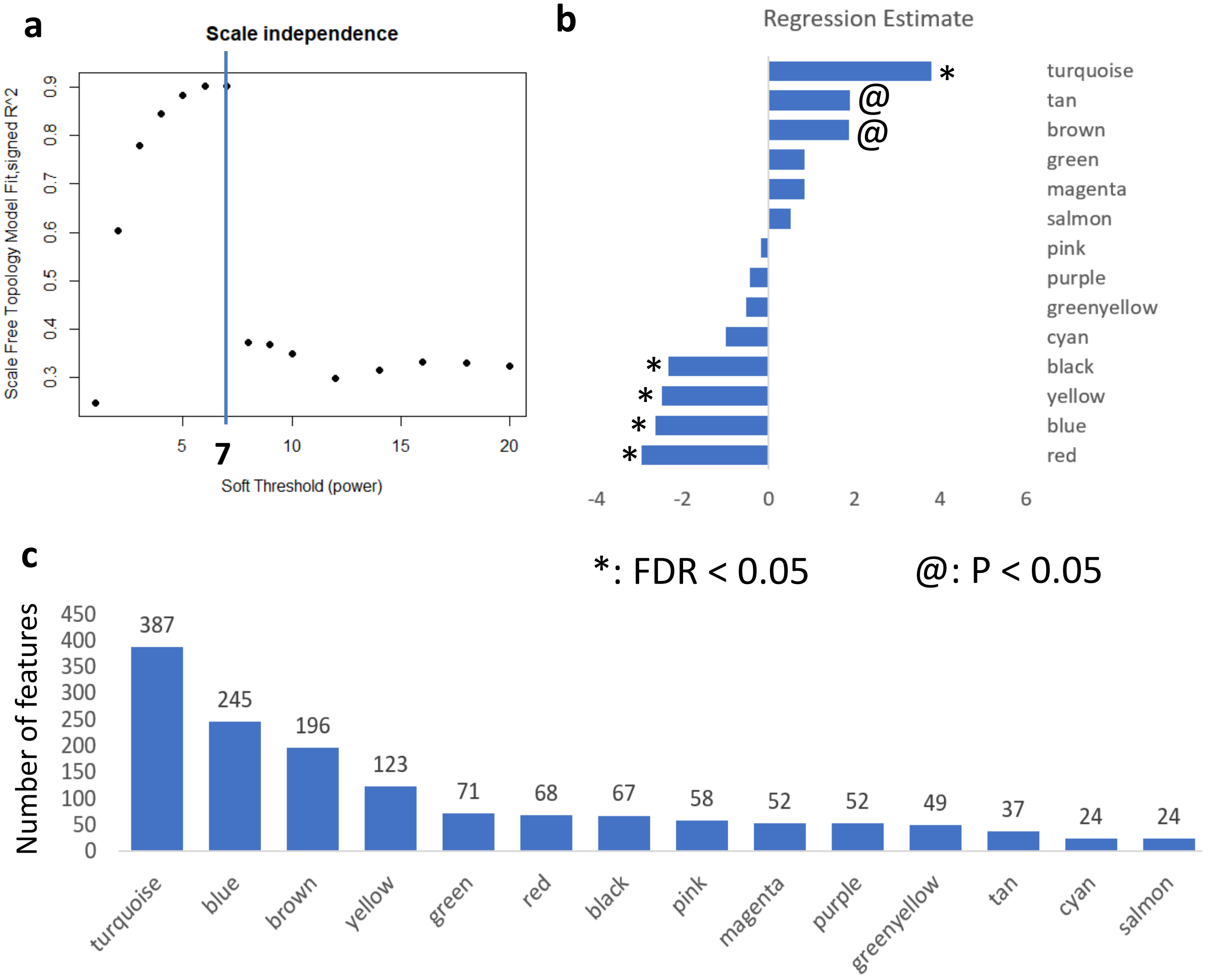

Figure S11. *Overview of the steps and the results of combined WGCNA from the three data layers*.
(a) shows the Scaled-Independence plot and the Scale-free topology fit and highlights the selection of the soft-power of 7 as it has the maximum scale-free nature for the network. (b) Shows the regression coefficients of the 14 modules obtained using Ordinary Least-square Regression for worse outcome (where in the outcomes were ranked as 1 for mild and moderate; 2 for severe and 3 for death). The modules with significant (Benjamini-Hochberg corrected FDR ≤ 0.05) and nominal associations (P ≤ 0.05) are also indicated. (c) Shows the sizes of the different modules in terms of the feature.

a

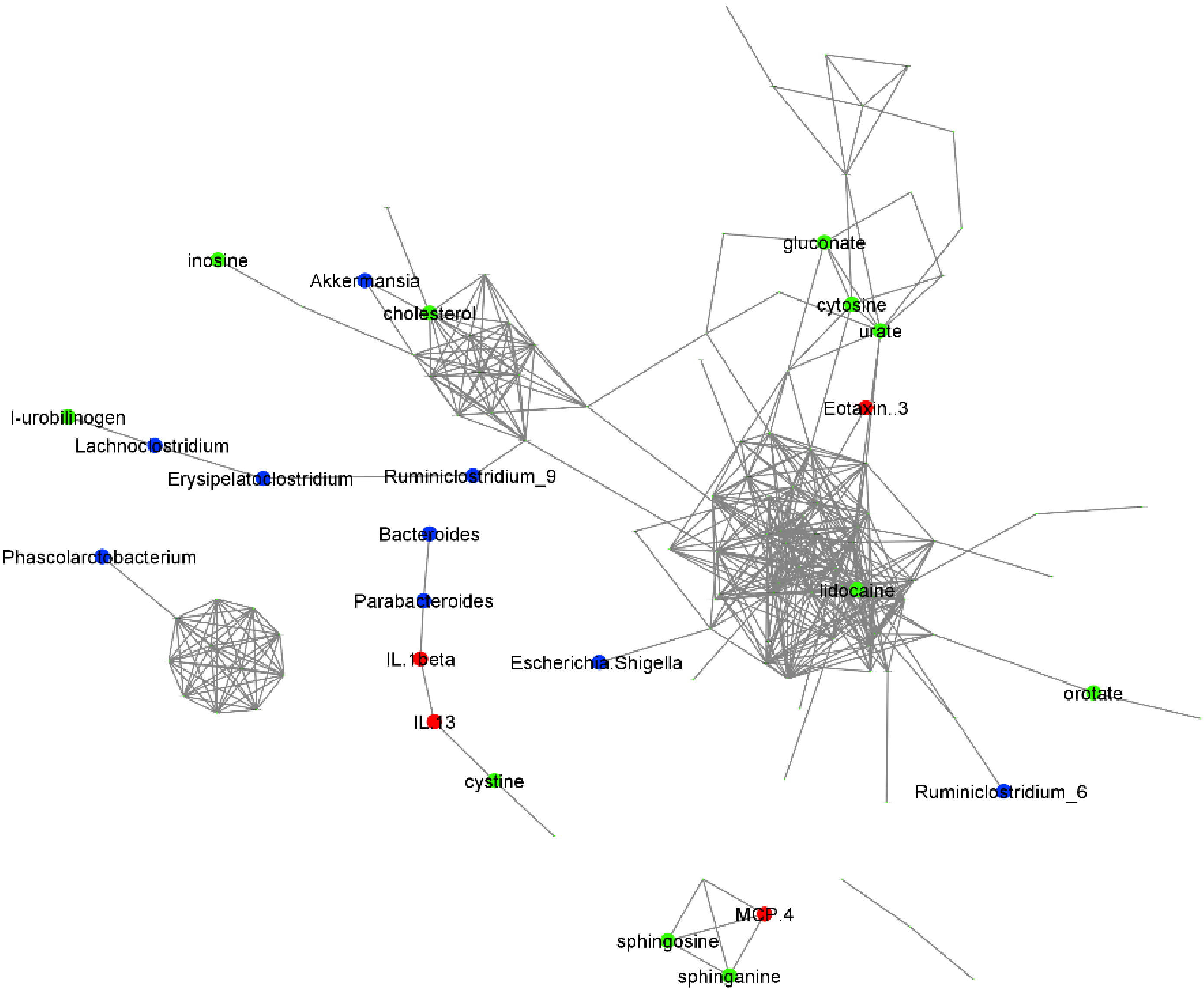

Figure S12. Modules showing nominal positive associations with severity and death. Positive association networks obtained for the features affiliated to (a) brown and (b) the tan module, using the ccrepe approach (Spearman correlation, iterations = 1000, p ≤ 0.01). Key taxa and metabolites are highlighted.

b

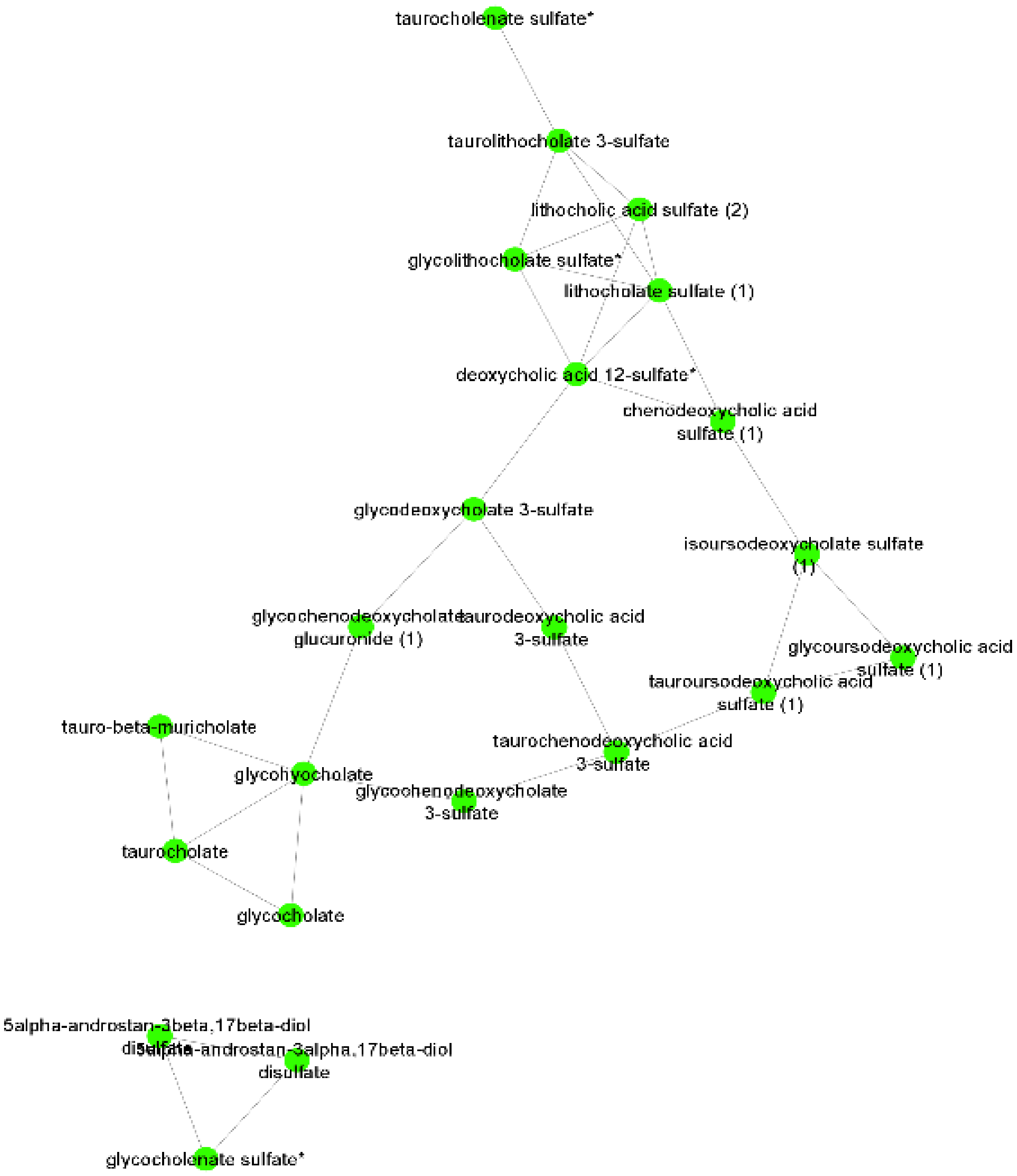

Figure S12. Modules showing nominal positive associations with severity and death (continued)
Positive association networks obtained for the features affiliated to (a) brown and (b) the tan module, using the ccrepe approach (Spearman correlation, iterations = 1000, $p \leq 0.01$). Key taxa and metabolites are highlighted.

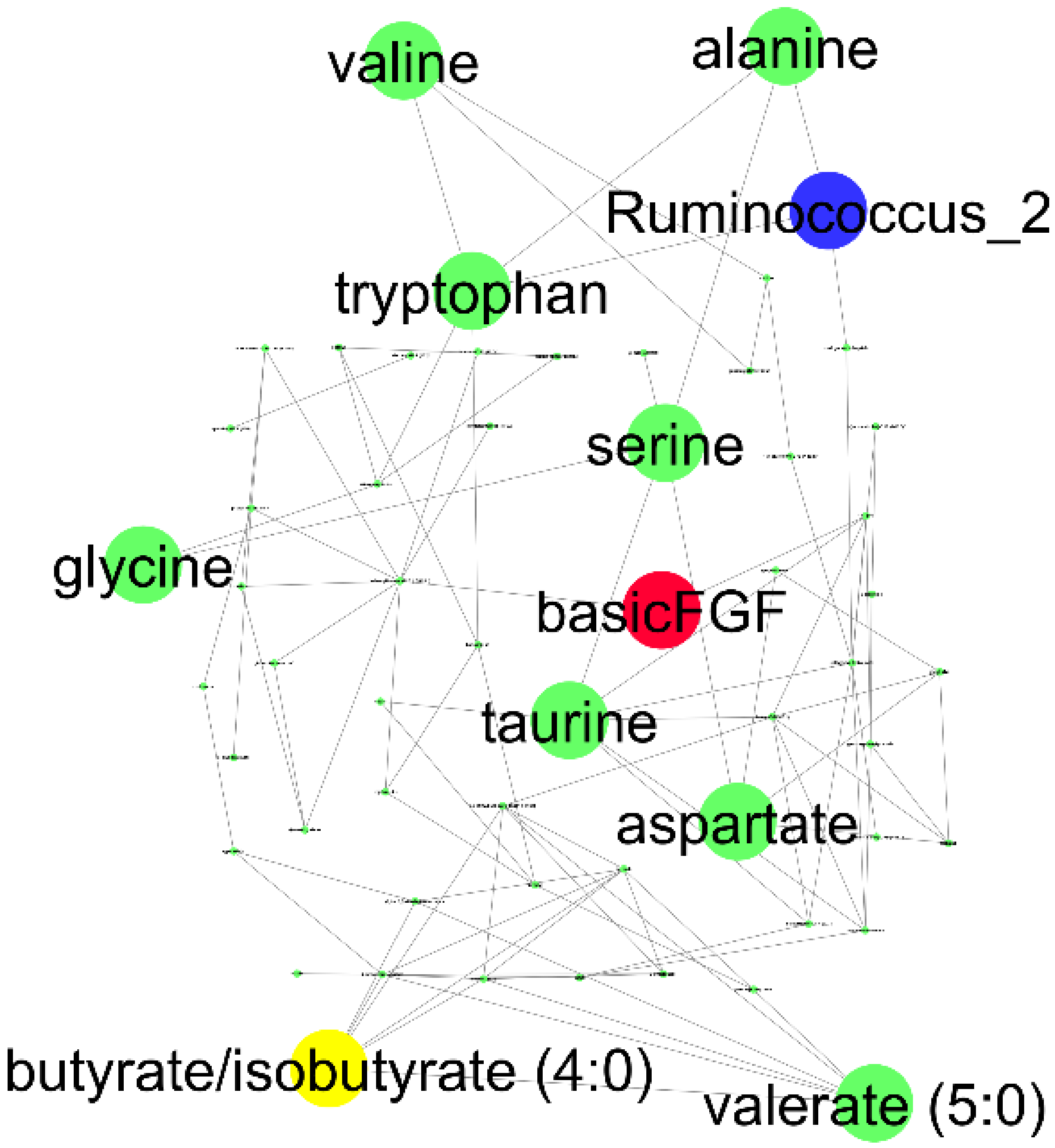

Figure S13. Association patterns within the 'red' module.
Positive association networks obtained for the features affiliated to the red module, using the ccrepe approach (Spearman correlation, iterations = 1000, p <= 0.01). Key taxa and metabolites are highlighted.

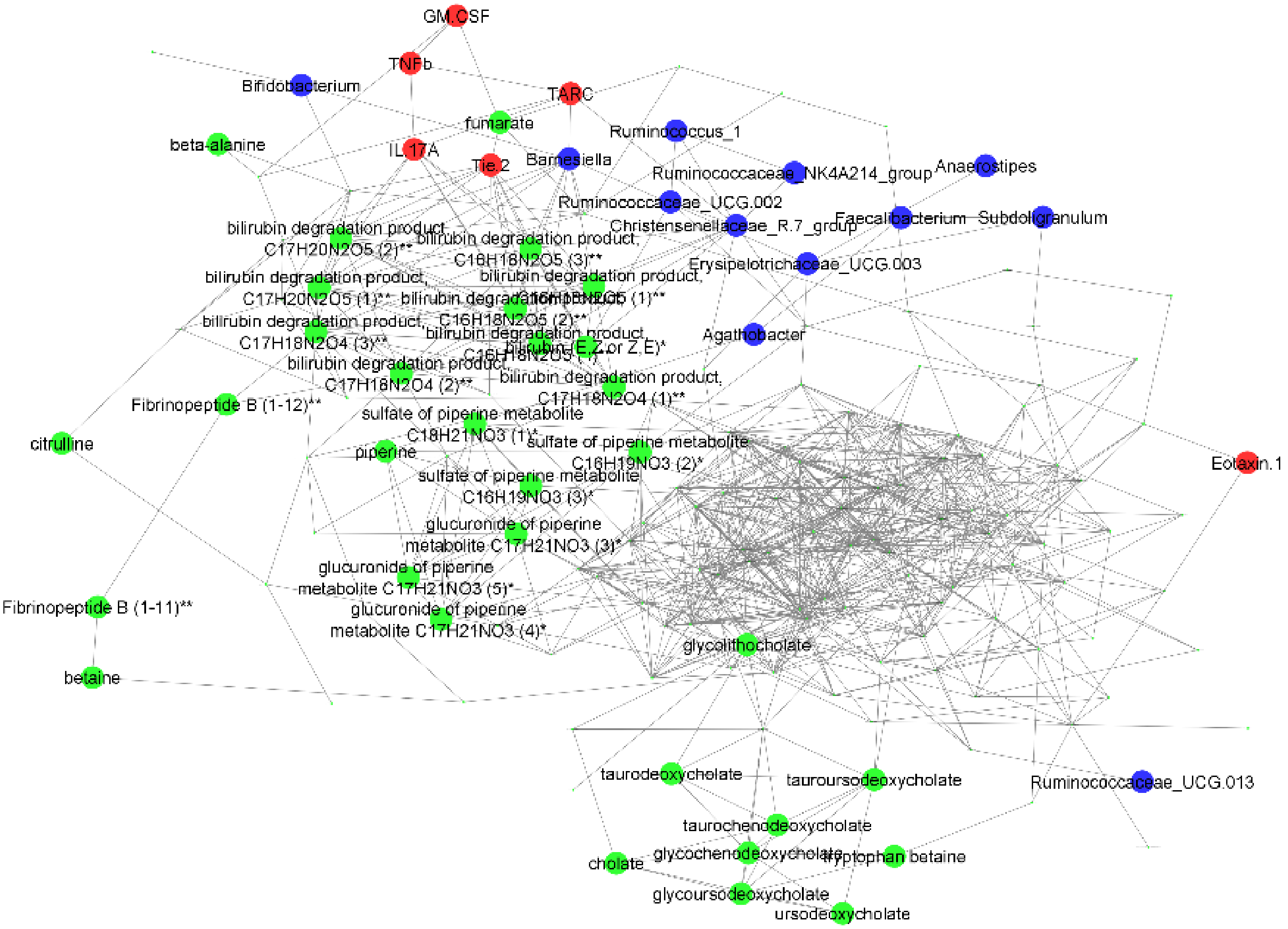

Figure S14. Association patterns within the 'blue module.
Positive association networks obtained for the features affiliated to the blue module, using the ccrepe approach (Spearman correlation, iterations = 1000, p <= 0.01). Key taxa and metabolites are highlighted.

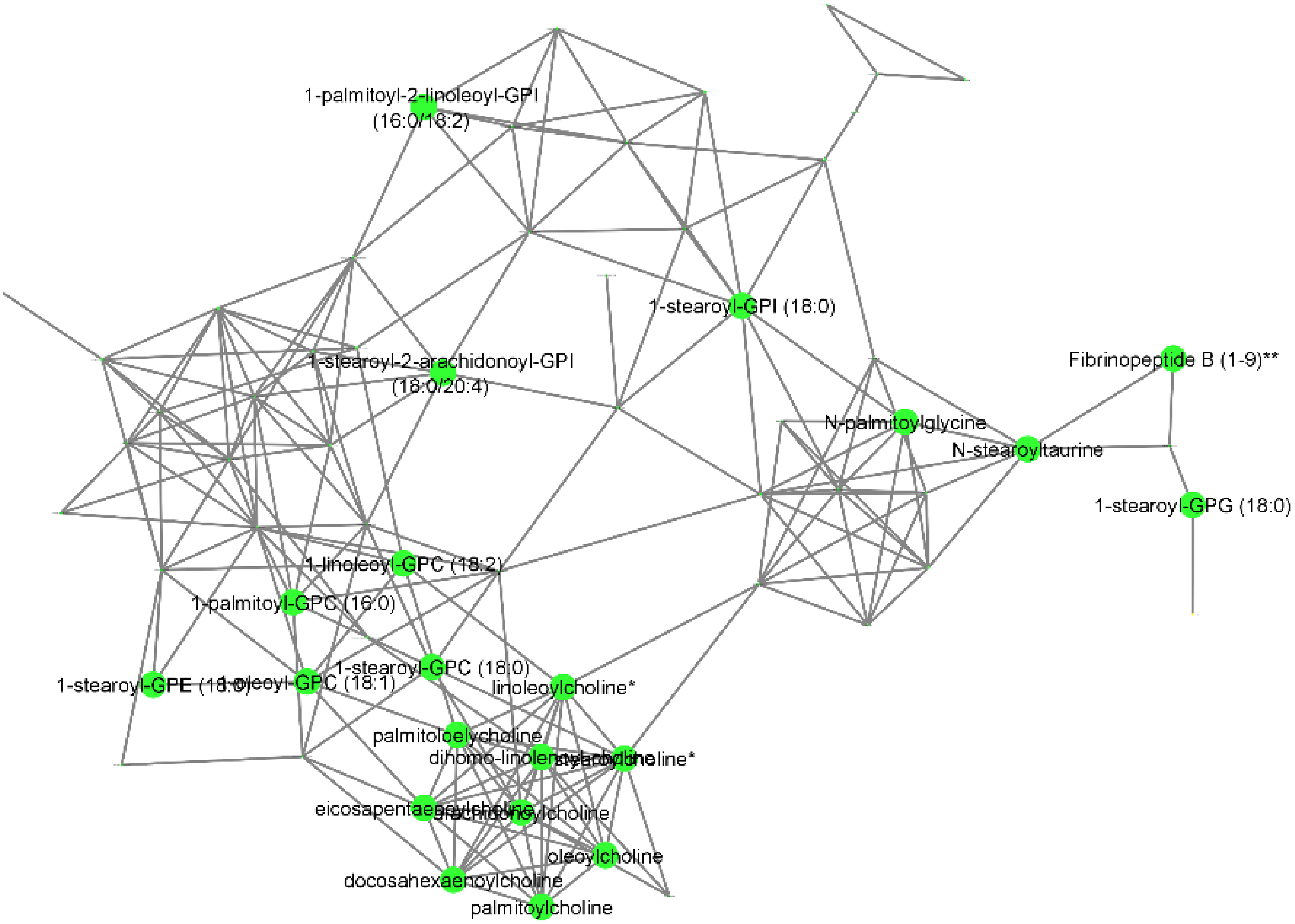

Figure S15. Association patterns within the 'black' module.
Positive association networks obtained for the features affiliated to the black module, using the ccrepe approach (Spearman correlation, iterations = 1000, p <= 0.01). Key taxa and metabolites are highlighted.

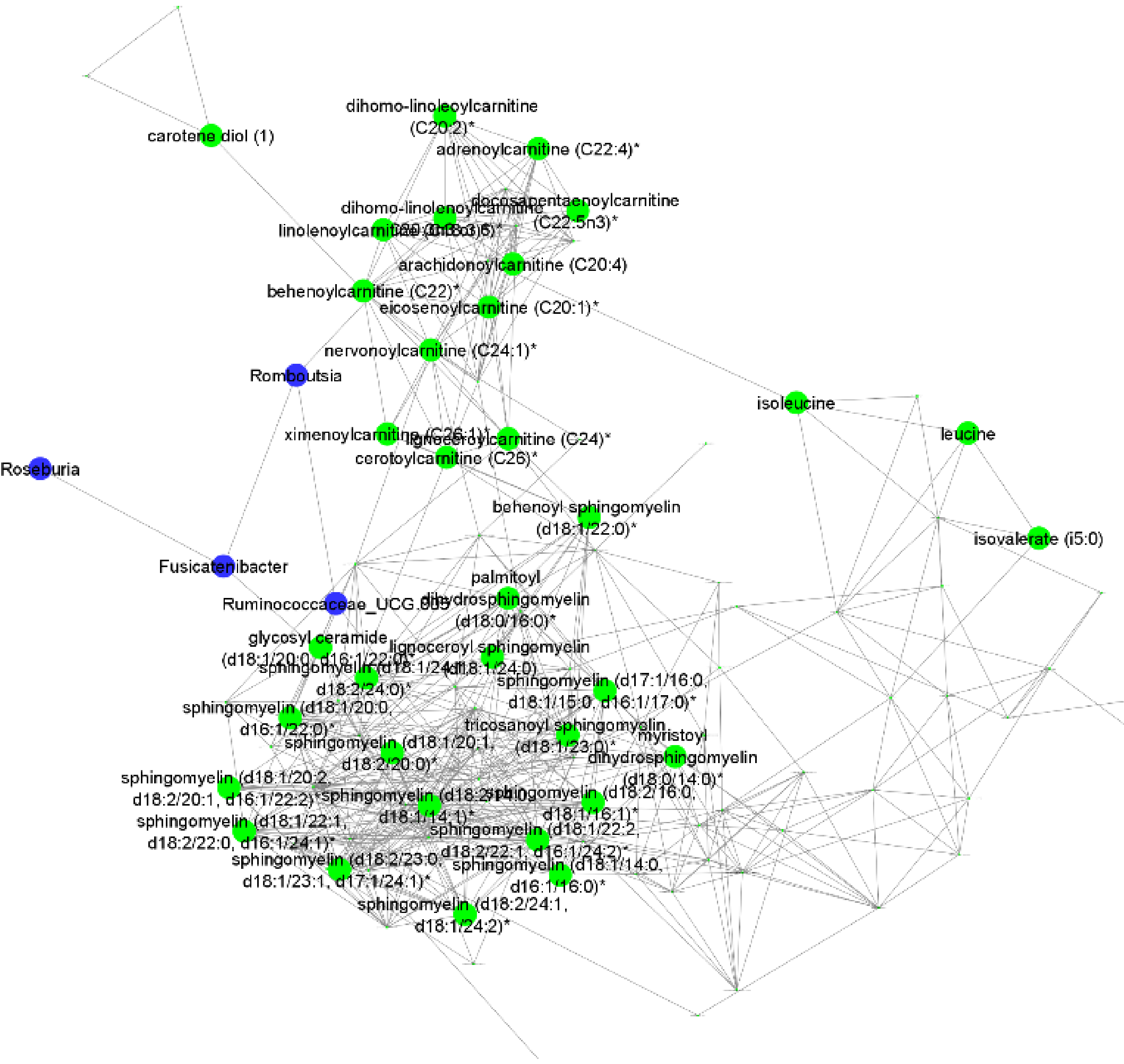

Figure S16. Association patterns within the 'yellow module.
Positive association networks obtained for the features affiliated to the yellow module, using the ccrepe approach (Spearman correlation, iterations = 1000, p <= 0.01). Key taxa and metabolites are highlighted.
